# IL10 deficiency promotes alveolar enlargement and lymphoid dysmorphogenesis in the aged murine lung

**DOI:** 10.1111/acel.13130

**Published:** 2020-03-14

**Authors:** Alla Malinina, Dustin Dikeman, Reyhan Westbrook, Michelle Moats, Sarah Gidner, Hataya Poonyagariyagorn, Jeremy Walston, Enid R. Neptune

**Affiliations:** ^1^ Pulmonary and Critical Care Medicine Johns Hopkins School of Medicine Baltimore MD USA; ^2^ Division of Geriatrics Johns Hopkins School of Medicine Baltimore MD USA; ^3^ Departments of Biology and Chemistry and Biochemistry Florida International University Miami FL USA

**Keywords:** alveolar epithelial cells, emphysema, IL10, lymphoid aggregates, macrophages, MMP12

## Abstract

The connection between aging‐related immune dysfunction and the lung manifestations of aging is poorly understood. A detailed characterization of the aging IL10‐deficient murine lung, a model of accelerated aging and frailty, reconciles features of both immunosenescence and lung aging in a coherent model. Airspace enlargement developed in the middle‐aged (12 months old) and aged (20–22 months old) IL10‐deficient lung punctuated by an expansion of macrophages and alveolar cell apoptosis. Compared to wild‐type (WT) controls, the IL10‐deficient lungs from young (4‐month‐old) mice showed increased oxidative stress which was enhanced in both genotypes by aging. Active caspase 3 staining was increased in the alveolar epithelial cells of aged WT and mutant lungs but was greater in the IL10‐deficient milieu. Lung macrophages were increased in the aged IL10‐deficient lungs with exuberant expression of MMP12. IL10 treatment of naïve and M2‐polarized bone marrow‐derived WT macrophages reduced MMP12 expression. Conditioned media studies demonstrated the secretome of aged mutant macrophages harbors reduced AECII prosurvival factors, specifically keratinocyte growth factor (KGF) and hepatocyte growth factor (HGF), promotes cell death, and reduces survival of primary alveolar epithelial cells. Compared to WT controls, aged IL10‐deficient mice have increased parenchymal lymphoid collections comprised of a reduced number of apoptotic cells and B cells. We establish that IL10 is a key modulator of airspace homeostasis and lymphoid morphogenesis in the aging lung enabling macrophage‐mediated alveolar epithelial cell survival and B‐cell survival within tertiary lymphoid structures.

## Graphical abstract

The loss of IL10 causes airspace enlargement and expansion of lymphoid aggregates in the aged murine lung. Enhanced alveolar epithelial cell death from reduced macrophage‐secreted prosurvival factors and increased MMP12 production contributes to the airspace phenotype. The expansion of altered lymphoid aggregates in the aged mutant lung features reduced cell apoptosis and reduced B‐cell content. IL10 is a key modulator of airspace homeostasis and lymphoid morphogenesis in the lung.

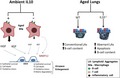

## 1 INTRODUCTION

Aging‐related lung disorders such as emphysema, pulmonary fibrosis, and serious pneumonias are costly and confer a significant disease burden on older adults (Murray and Chotirmall (2015)). Known contributory mechanisms include oxidative stress, impaired tissue repair, and compromised host defense or immunosenescence. Unfortunately, the nexus connecting aging‐related lung disorders and immunosenescence is unknown. Aberrant lymphopoiesis, impaired lymphocyte turnover, exaggerated immunoglobulin production, and compromised establishment of protective immunity punctuate immunosenescence (Calvi et al., 2011; Nikolich‐Zugich, 2018). If an aging immune system promotes the development of an aging lung phenotype, genetically defined murine models which recapitulate either systemic or lung‐specific immunosenescence might be especially informative. Here, we use a model of accelerated aging, chronic inflammation, and frailty, the IL10‐deficient (IL10^−/−^) mouse, to further dissect the lung‐specific consequences of immunosenescence(Walston et al., 2008).

IL10, a well‐studied TH2 cytokine produced by multiple immune cells, plays a complex role in infectious, autoimmune, or inflammatory injuries during aging by exerting both anti‐inflammatory and pro‐inflammatory effects that are context dependent (De Beaux, Maingay, Ross, Fearon, & Carter, 1995; Itoh & Hirohata, 1995; Ouyang & O'Garra, 2019). IL10 inhibits T‐cell activation, macrophage‐accessory cell interactions, Th1 responses but also induces B‐cell differentiation, immunoglobulin production, and Th2 responses(Rennick, Davidson, & Berg, 1995). Population surveys and murine models show reduced systemic IL10 levels in older adults associated with adverse cardiovascular risks and shortened lifespan permitting the connection between aging‐associated immunosenescence and the loss of IL10 activity (Belloni et al., 2010; Dimitrijevic et al., 2014; Khabour & Barnawi, 2010; Van Den Biggelaar et al., 2004). Compromised production of IL10 with secondary impairment of immunoregulatory functions develops with aging (Duggal, Upton, Phillips, Sapey, & Lord, 2013; Williams, Jose, Brown, & Chambers, 2015). Walston found that features of frailty, specifically sarcopenia, chronic elevation in mediators of inflammation, and early mortality developed with increasing age in the IL10^−/−^ mouse implicating a possible causal connection between immunosenescence and systemic manifestations of aging (Walston et al., 2008; Westbrook et al., 2017). IL10^−/−^ mice display a complex susceptibility to lung infections including more severe RSV infection and corynebacterial pneumonia but reduced severity of influenza infection (Jeong, Won, Kim, Cho, & Choi, 2009; Loebbermann et al., 2012; McKinstry et al., 2009). While low IL10 levels associate with asthma, COPD, and active smoking, elevated levels contribute to postbronchiolitis wheezing (Bont et al., 2000; Borish et al., 1996; Takanashi et al., 1999). These findings suggest that the effects of altered IL10 levels in the lung are context‐dependent and cannot be predicted based on the fundamental properties of the cytokine.

We sought to determine whether IL10 deficiency, as a model of immunosenescence, impairs lung homeostasis during aging using IL10^−/−^ mice. We found that IL10^−/−^ mice develop accelerated age‐related airspace enlargement as well as age‐progressive lung lymphoid aggregates (LAs, sometimes described as bronchus‐associated lymphoid tissue (BALT) (Randall, 2010). These suggest that the exaggerated immunosenescent milieu of IL10 deficiency creates a hospitable environment for reduced alveolar epithelial survival, altered lymphoid dynamics, and macrophage infiltration culminating in lymphoid dysmorphogenesis and emphysema.

## 2 RESULTS

### 2.1 Role of IL10 in aging‐related airspace injury

**Figure 1:**
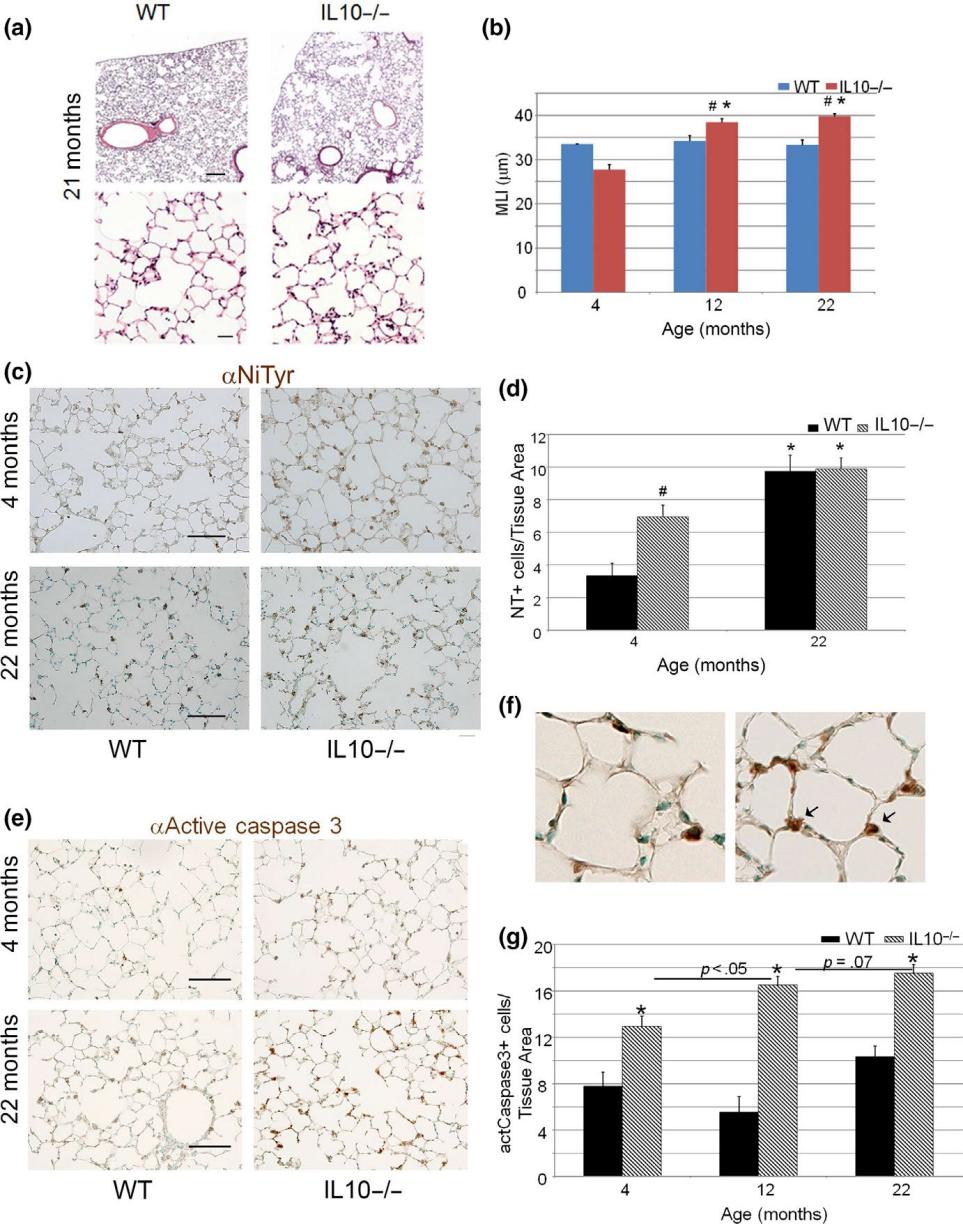
Progressive airspace enlargement and alveolar injury in IL10‐deficient mice. (a) Representative hematoxylin and eosin staining of mouse lungs from aged (21‐month‐old) WT and IL10‐deficient mice (*N* = 5–8 per condition). Top 10×, Bottom 40× magnification. Scale bar: Top‐150 µm, Bottom 50 µm. (b) Quantitative alveolar morphometry of lungs from WT and IL10‐deficient lungs at designated ages (*N* = 5–9 mice per genotype per age). (c) Representative nitrotyrosine immunohistochemistry of WT and IL10‐deficient lungs at designated ages (*N* = 4–5 mice per genotype per age). Scale bar 100 µm. (d) Quantitative immunohistochemistry of nitrotyrosine staining normalized to tissue area of murine lungs of designated age and genotype (*N* = 4–5 mice per genotype per age). (e) Representative active caspase 3 immunohistochemistry of WT and IL10‐deficient lungs at designated ages (*N* = 4–5 mice per genotype per age). Scale bar 75 µm. (f) Higher power view of active caspase 3 immunohistochemistry of WT and mutant mice showing localization to alveolar epithelial type II cells (note arrows). (g) Quantitative immunohistochemistry of active caspase 3 staining of WT and mutant lungs normalized to tissue area. (*N* = 4–5 mice per genotype per age). *p < .01 compared with young mice of the same genotype, ^#^ *p *< .01 compared with age‐matched WT mice. Tissue Area‐pixels‐squared

Morphometric analysis revealed a 48% age‐related progressive increase in airspace dimension in the IL10‐deficient lungs that was significantly greater than that observed in WT mice (Figure 1a,b). Because oxidative stress associates with emphysema and IL10 deficiency, we examined markers of oxidant injury in the aged mutant lung (Huet et al., 2013; Kinzenbaw, Chu, Pena Silva, Didion, & Faraci, 2013). Nitrotyrosine staining, reflecting nitrosative and oxidative stress, showed a significant increase in oxidative injury in the young IL10^−/−^ lung compared with WT mice (Figure 1c,d). With aging, there was a greater increase in oxidative stress in the WT lung resulting in a similar level of oxidative stress in both genotypes at 22 months of age. Since enhanced intestinal epithelial cell death is associated with IL10 deficiency, and alveolar epithelial and endothelial cell death can cause airspace enlargement, we examined these compartments using active caspase 3 immunohistochemistry (Mizushima et al., 2013; Tuder, Petrache, Elias, Voelkel, & Henson, 2003). We observed a progressive increase, in alveolar epithelial cell apoptosis in aging IL10^−/−^ lungs compared with age‐matched WT controls (Figure 1e–g). Of note, a more significant increase in epithelial apoptosis developed in the aging mutant lung parenchyma but not the WT lung, suggesting that reduced lung cell survival might contribute to the airspace enlargement. IL10 acts by binding to a transmembrane glycoprotein receptor complex containing both IL10ra and IL10Rb (Shouval et al., 2014). Whereas IL10Ra is specific for IL10, IL10Rb can engage multiple cytokines when partnered with alternative co‐receptors. To determine whether the reduced alveolar epithelial cell survival was secondary to a direct IL10‐mediated effect, we first measured the levels of IL10 receptors by RT–PCR in alveolar epithelial type II cells isolated from adult WT C57Bl/6 mice. We found very low levels of the IL10Ra mRNA, the specific component of the IL10R, in AECII cells (Figure S1a). Moreover, no IL10Ra was detected in AECII cells by Western blot or immunohistochemistry (Figure S1b and data not shown). These data suggest an indirect protective role of IL10 in alveolar epithelial cells.

### 2.2 IL10 and organ‐specific innate immune populations in aged mice

**Figure 2:**
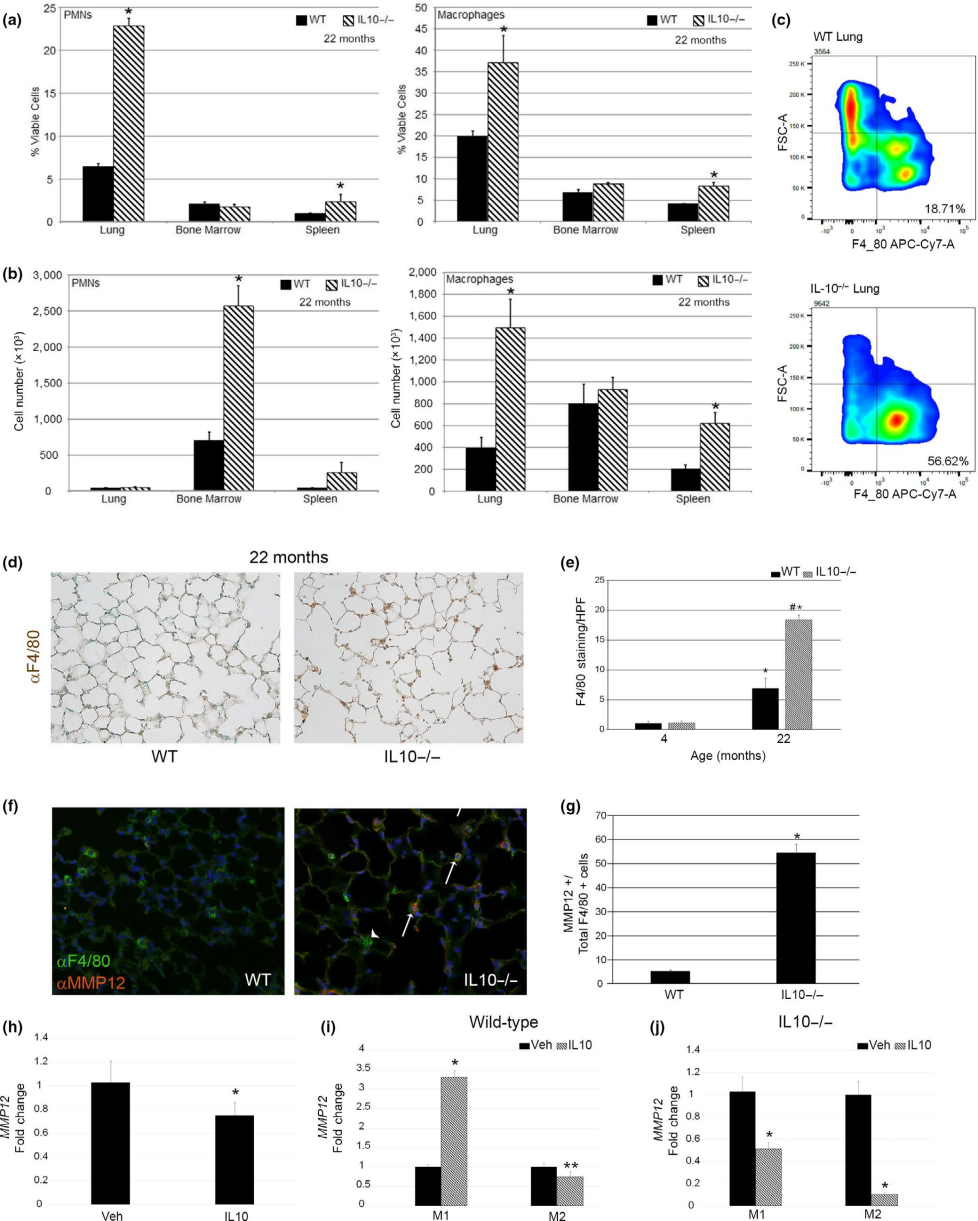
Macrophage expansion and increased metalloprotease production in IL10‐deficient lungs. (a) Flow cytometry quantitation of neutrophils and macrophages in aged WT versus IL10‐deficient lungs, bone marrow, and spleen. (*N* = 5–8 mice/genotype). (b) Absolute quantitation of PMNs and macrophages in noted groups. (c) Representative flow cytometry of macrophages in lungs of WT versus IL10‐deficient lungs by F4/80 staining. (d) Representative F4/80 immunohistochemical staining of aged murine lungs of designated genotypes. 20× images. (e) Quantitative immunohistochemistry of F4/80 staining of young and aged WT and IL10‐deficient lungs. *N* = 3–5 mice per genotype per age. (f) Representative immunofluorescent costaining for F4/80 and MMP12 in aged murine lungs of designated genotypes. Arrowhead denotes macrophage not expressing MMP12. Arrows denote macrophages expressing MMP12. G. Quantitative immunohistochemistry of F4/80 and MMP12 costaining. *N* = 3–5 mice per genotype per age. Data are representative of 4–5 independent experiments

Innate cell abundance and activity in COPD patients and cigarette smoke‐exposed animal models directly contribute to parenchymal lung injury, altered lung function, and durable architectural and physiologic deficits beyond active smoke exposure (Barnes, 2017; Ghorani, Boskabady, Khazdair, & Kianmeher, 2017). By flow cytometry, we found enhanced neutrophilic and monocyte/macrophage inflammation in the mutant lungs and spleen (Figure 2a–c). The 3.7‐ and 3.0‐fold increase in absolute macrophage abundance in the lungs and spleen, respectively, but not bone marrow of mutant mice suggests in situ expansion. IL10 induces a potent transcriptional response in monocyte/macrophages resulting in broad cytokine dysregulation, enhanced phagocytosis but reduced pathogen killing, and attenuated motility (Buchwald, Geerdes‐Fenge, Vockler, Ziege, & Lode, 1999; Vicioso et al., 1998; de Waal Malefyt, Abrams, Bennett, Figdor, & de Vries, 1991). Monocytes and macrophages exert much of the immunosuppressive effects of IL10 in the lungs(Wissinger, Goulding, & Hussell, 2009). These findings suggest that both systemic‐ and organ‐specific changes in innate immunity accompany IL10 deficiency.

### 2.3 Loss of IL10 enables lung macrophage infiltration and increases metalloprotease expression

A central pathogenetic paradigm of COPD/emphysema is that the expansion and activation of resident alveolar macrophages leads to the triggering of an inflammatory cascade with metalloprotease liberation culminating in alveolar wall destruction and emphysema (Barnes, 2016; Vlahos & Bozinovski, 2014). We assessed macrophages in situ by immunohistochemical staining and found markedly enhanced infiltration in the aged IL10^−/−^ airspace compared with young mutant mice and age‐matched WT controls (Figure 2d,e). This is consistent with the known resident macrophage expansion in the intestines of IL10^−/−^ mice (Zigmond et al., 2014). We also found a 10‐fold increase in macrophage MMP12 expression in the lungs of mutant aged mice but not mutant young mice supporting a contribution of MMP12 to the observed airspace enlargement (Figure 2f,g and data not shown). We saw no difference in MMP2 and MMP9 zymography between aged WT and mutant lungs (Figure S2a,b). MMP12 expression and activity were also not different in the whole lungs of aged mutant mice but did show a trend toward increase (Figure S2c,d).

We examined IL10R expression in BMDMs and found no difference in IL10Ra expression between WT and mutant macrophages but did see a significant reduction in IL10Rb expression in mutant cells (Figure S3).

Using a co‐culture system, John‐Schuster recently showed that B‐cell line‐derived IL10 can induce MMP12 expression in MHS cell line (John‐Schuster et al., 2014). When we treated aged WT macrophages with IL10, we observed a 31% reduction in MMP12 expression (Figure 2h). In an acute lung injury model, Aggarwal observed that macrophage subsets exhibit different autocrine IL10 production (Aggarwal et al., 2014). (Marker features of these populations are described in Figure S4). Upon polarizing BMDMs, we observed that exogenous IL10 increased MMP12 expression by threefold in WT M1 cells but reduced MMP12 expression in WT M2 cells by 33% (Figure 2i). We examined whether IL10^−/−^ macrophage subsets show altered responsiveness to IL10 by treating M1‐ and M2‐biased mutant BMDMs with IL10 and measuring MMP12 expression. Mutant M1 cells demonstrated 50% downregulation, and M2 cells showed 90% downregulation of MMP12 (Figure 2j). Collectively, these data suggest that the cytokine IL10 tempers MMP12 expression in the murine lung via M0 and M2 cells potentially suppressing elastase‐driven airspace enlargement.

### 2.4 Secretome of aged IL10‐deficient macrophages reduces alveolar epithelial survival and enhances apoptosis and oxidative stress

**Figure 3:**
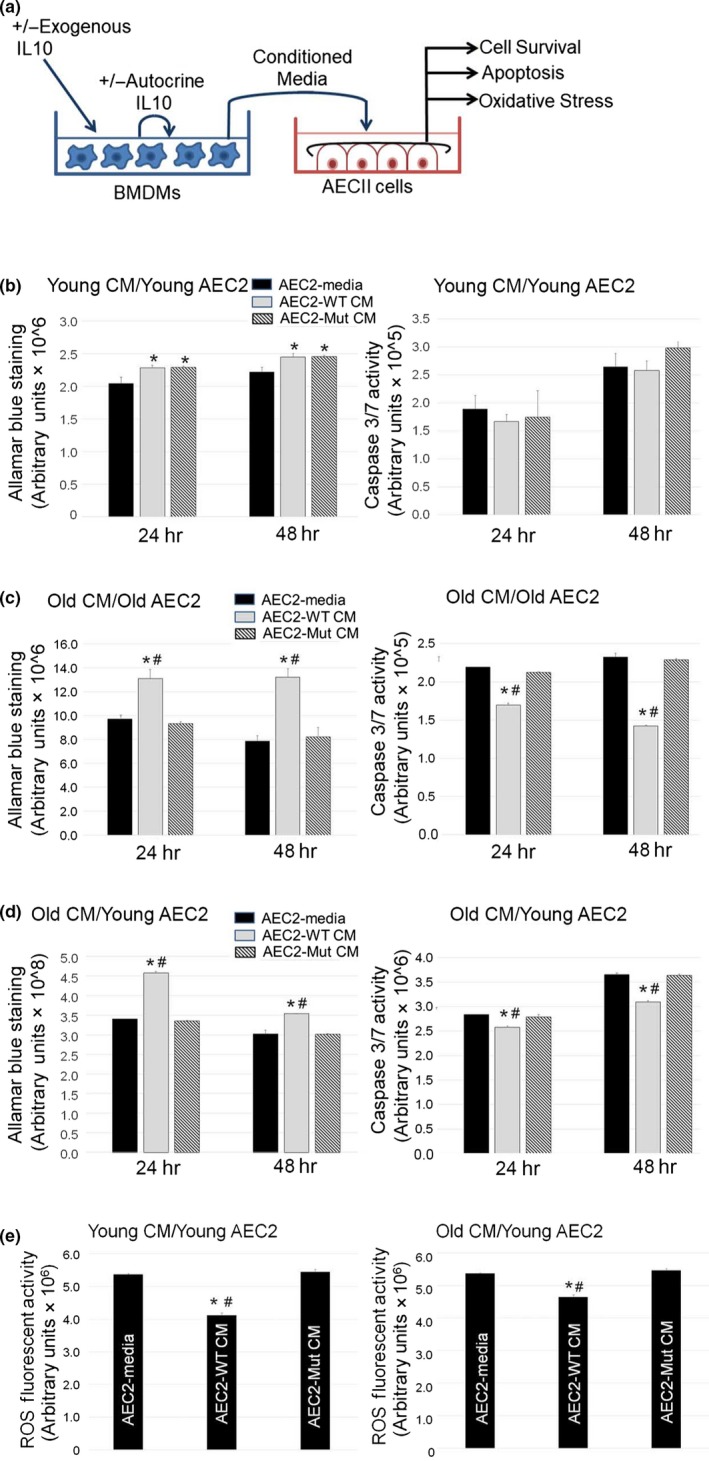
IL10‐dependent macrophage‐epithelial prosurvival secretome. (a) Schematic for macrophage CM studies to identify epithelial prosurvival effects of IL10 exposure. A dilution series of CM from WT and IL10‐deficient bone marrow‐derived macrophages (BMDMs) was obtained. Isolated murine age‐specified alveolar epithelial cells (AECIIs) were exposed to equivalent dilutions of CM from young or aged macrophages. Cell survival, apoptosis, and oxidative stress were assessed at different time points. (b) Effect of exposure of young AECIIs to CM from young WT and IL10‐deficient mice on survival and caspase 3‐mediated cell death. (c) Effect of exposure of old AECIIs to CM from old WT and IL10‐deficient mice. (d) Effect of exposure of young AECIIs to CM from old WT and IL10‐deficient mice. (e) Effect of exposure of young AECIIs to CM from young and old WT and IL10‐deficient mice on oxidative stress. Studies represent BMDM harvests from 4 to 5 mice of specified genotypes and ages. All performed in triplicates or quadruplicates

Since IL10Ra was not expressed on AECII cells, we explored whether the secretory program of macrophages, in response to IL10, impacts AECII survival and function. We generated BMDMs from aged and young WT and IL10^−/−^ mice and evaluated selective effects of macrophage‐conditioned media (CM) on primary murine alveolar epithelial cells, AECII (Figure 3a). We found that CM from young mutant BMDMs did not reduce AECII survival compared with CM from young WT mice (Figure 3b). However, CM from aged mutant BMDMs conferred reduced survival and increased apoptosis of both young and aged AECIIs (Figure 3c,d). These results suggest that altered IL10‐deficient macrophage secretome is a driver of AECII death and may contribute to airspace enlargement in the aged IL10‐deficient lung.

We examined whether IL10‐deficient macrophages contributed to the enhanced oxidative stress observed in the alveolar compartment of the lung. Exposure of young AECII cells to CM from young WT macrophages conferred a 25% reduction in oxidative stress observed with exposure to CM from young mutant macrophages (Figure 3e). Similarly, young AECII cells exposed to CM from old WT macrophages showed only a 12% reduction in oxidative stress compared with CM from aged mutant macrophages. Despite elevated levels of ROS overall, CM from aged WT but not mutant BMDMs were still able to attenuate oxidative injury of aged AEC2 cells (Figure S6). While attenuated, the antioxidant effects of the WT macrophage secretome remained despite aging.

### 2.5 IL10‐mediated macrophage secretome reveals KGF and HGF as candidate epithelial prosurvival factors

**Figure 4:**
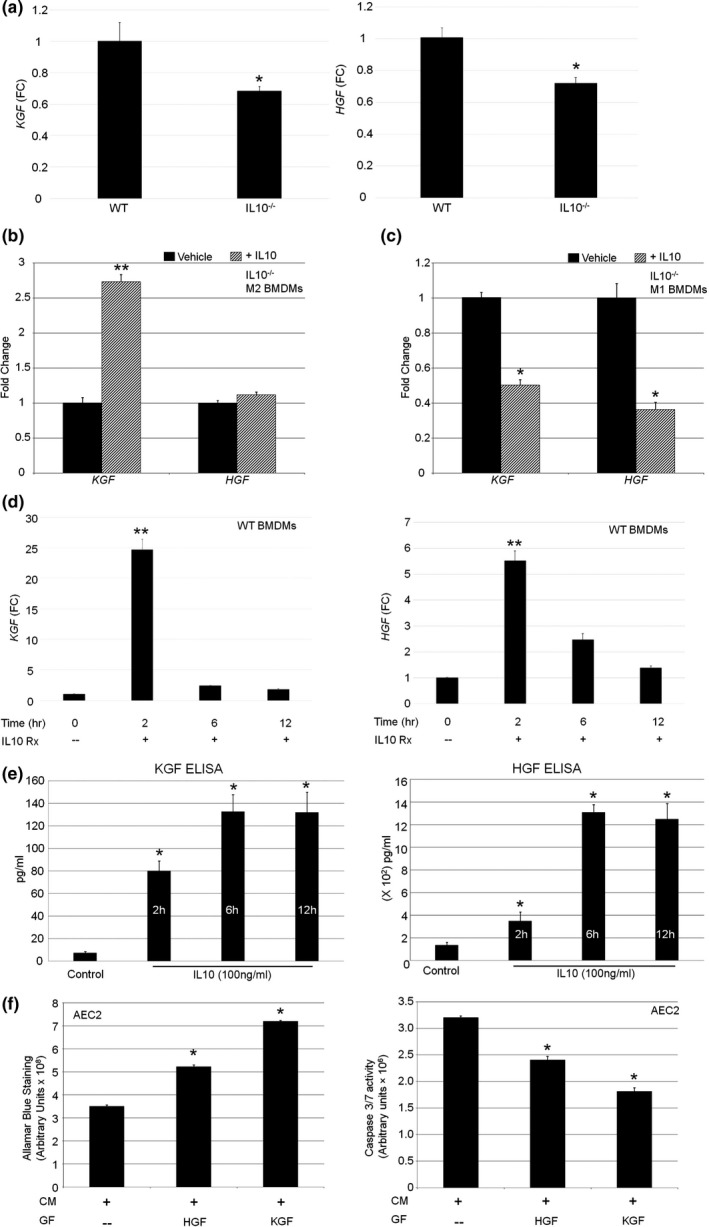
IL10‐dependent expression of prosurvival growth factors by macrophages. (a) Relative expression by qPCR of indicated mRNAs in BMDMs isolated from aged (22‐month‐old) WT and IL10‐deficient mice. *N* = 4–5 mice per genotype. (b) IL10‐dependent expression of KGF and HGF by M2‐polarized BMDMs from IL10‐deficient mice. *N* = 5 mice. (c) IL10‐dependent expression of KGF and HGF by M1‐polarized BMDMs from IL10‐deficient mice. *N* = 5 mice. (d) Time course of IL10 induction of KGF (left) and HGF (right) by IL10 in young WT BMDMs. (e) Time course of ELISA‐quantified antigenic KGF (left) and HGF (right) in CM from IL10‐treated young WT BMDMs. (f) Effect of HGF or KGF supplementation of BMDM‐CM from aged IL10‐deficient mice on AEC2 survival and apoptosis. *p < .01 compared with control condition. FC‐fold change

Both HGF and KGF are established epithelial survival factors known to promote alveolar repair in injury states (Adamson & Bakowska, 1999; Plantier et al., 2007). We previously reported that HGF signaling is required for normal airspace formation in a murine model (Calvi et al., 2013). Under basal conditions, aged IL10^−/−^ BMDMs exhibited reduced HGF and KGF expression compared with WT BMDMs (Figure 4a). Since M2 macrophages are known to be most responsive to IL10, we examined the IL‐10‐mediated transcriptome of middle‐aged IL10^−/−^ M1 and M2‐polarized BMDMs and found a threefold increase in KGF but not HGF in M2 subtypes (Figure 4b). IL10‐treated mutant M1 macrophages showed reduced KGF and HGF expression (Figure 4c). WT aged BMDMs treated with IL10 showed a twofold induction of KGF and a fivefold induction of HGF by qPCR (Figure 4d). By ELISAs, KGF was induced 18‐fold and HGF 10‐fold with IL10 treatment (Figure 4e). Finally, using the CM approach, we also found that the addition of KGF or HGF to aged mutant BMDM‐CM improved AECII cell survival and apoptosis (Figure 4f). These findings implicate both HGF and KGF as components of the epithelial prosurvival secretome of WT macrophages which is compromised in an IL10^−/−^ or aging context, supporting findings in Figure 3.

### 2.6 Lymphoid Aggregates Develop in the Lungs of IL10‐deficient Mice

**Figure 5:**
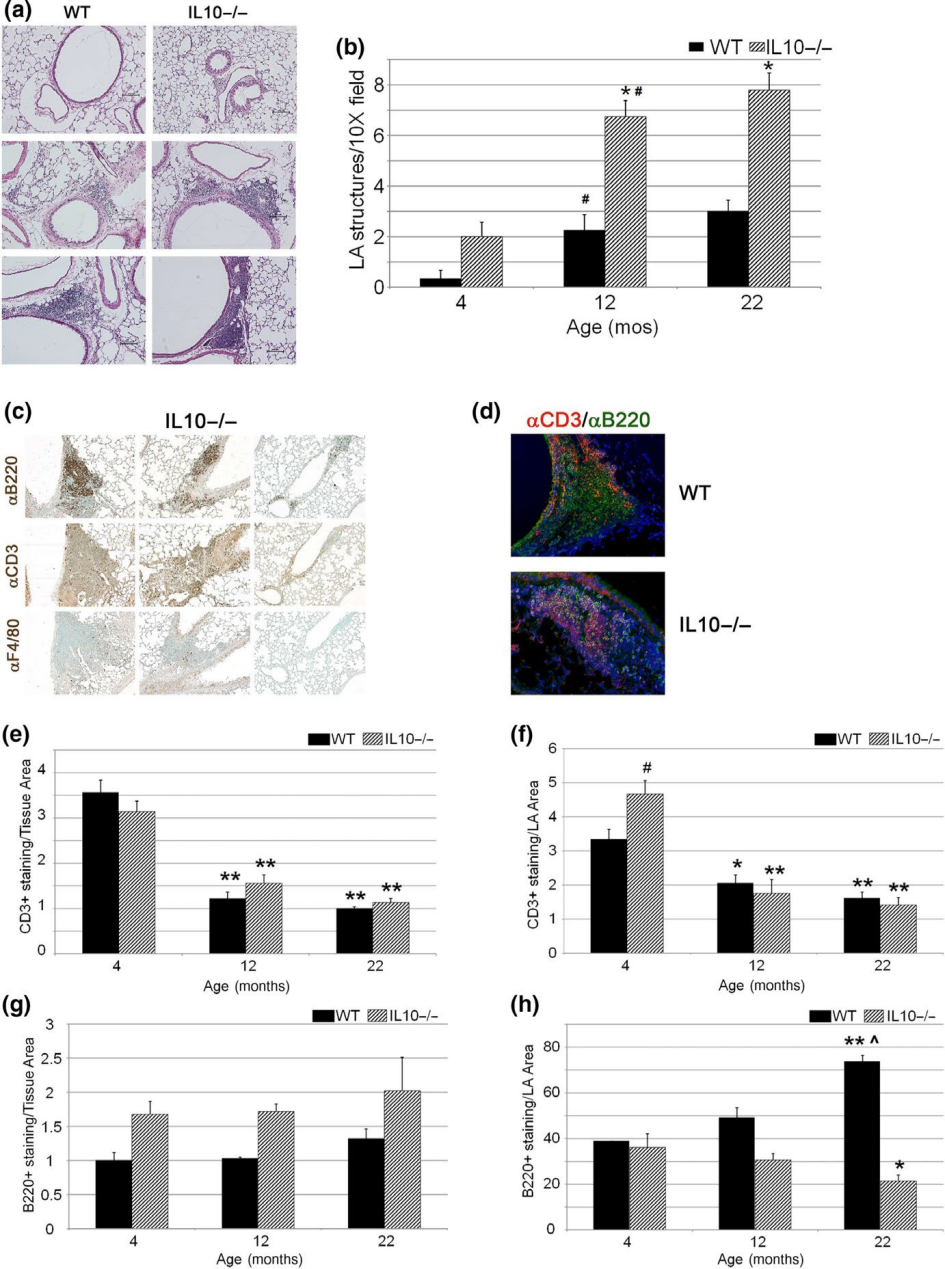
Age‐related lymphoid aggregate formation in IL10‐deficient mice. (a) Histologic changes in bronchovascular areas of 4‐month‐old, 12‐month‐old, and 22‐month‐old WT and IL10‐deficient lungs. Scale bar 150 µm, top, 100 µm, center, and bottom. (b) Quantitation of LA structures in WT and mutant mice at noted ages. (c) Immunohistochemical staining for B cell (B220), T cell (CD3), and macrophage markers in LAs of aged mutant lungs. (d) Immunofluorescent costaining for B‐cell and T‐cell representation in LAs of WT and IL10‐deficient aged lungs. (e) Quantitative IHC staining of T cells (CD3‐staining) in alveolar compartment from WT and IL10‐deficient lungs. (f) Quantitative IHC staining of T cells in LAs from WT and IL10‐deficient lungs. (g) Quantitative IHC staining of B cells (B220‐staining) in alveolar compartment from WT and IL10‐deficient lungs. (h) Quantitative IHC staining of B cells in LAs from WT and IL10‐deficient lungs. *N* = 5–8 mice per group. *p < .05, **p < .01 compared with 4‐mo‐old genotype‐matched mice, ^#^ *p* < .05 compared with age‐matched WT mice, ^*p* < .01 compared with 12‐month‐old genotype‐matched mice. Tissue Area‐ pixels‐squared

Lung lymphoid aggregates develop with aging, selected lung infections, and in certain autoimmune disorders (Foo & Phipps, 2010). Since IL10^−/−^ mice display intestinal inflammatory lesions with lymphoid infiltration, we considered whether a similar lesion exists in the lungs of mutant mice (Kuhn, Lohler, Rennick, Rajewsky, & Muller, 1993). We find a marked increase in number and size of airway lymphoid aggregates (LAs) in the 4‐month‐old mutant lung (Figure 5a,b; Figure S7). The abundance of these structures increases in the 12‐month‐old and 20‐ to 22‐month‐old mutant mice with evidence of perivascular involvement in the many aged mice. A survey of inflammatory cell composition within the LAs of young IL10^−/−^ mice reveals a large component of B220‐staining B‐lymphoid lineage cells and a much smaller representation of CD3‐ and F4/80‐staining cells, representing mature T cells and alveolar macrophages, respectively (Figure 5c). A similar representation of cell types was evident in the WT aged lungs (Figure S8). Immunofluorescent costaining for B cells and T cells reveals an architecture of airway LAs in WT lungs reminiscent of BALT structures with central B‐cell distribution and peripheral T cells but a loss of organization in the mutant lungs (Figure 5d) (Foo & Phipps, 2010). When we quantified lymphocyte abundance in these structures and in the lung parenchyma, we observed dynamic changes with genotype and aging. Although young IL10^−/−^ mice showed increased T‐cell abundance in LA structures compared to WT mice, T‐cell representation in the airspace and the LA structures decreased with aging in both genotypes (Figure 5e,f). By contrast, B‐cell abundance in LAs increased in the aging WT lung but fell in the IL10^−/−^ lung (Figure 5g,h). The expansion in LA formation and mean LA area in the aged IL10^−/−^ lung were accompanied by reduced B (B220+ or CD19+)‐ and T‐cell (CD3+) composition, suggesting that the mutant milieu supports the relative expansion of nonlymphoid but CD45‐positive cell populations. No significant staining for NK cell or dendritic cell markers was found in the LAs of the mutant mice (data not shown).

### 2.7 Altered lymphocyte abundance in aged IL10‐deficient organs

To determine whether there are lung‐specific changes in the adaptive immune milieu of aged IL10‐deficient lungs, we performed flow cytometry on lungs, bone marrow, and spleen of 22‐month‐old mutant and control mice. We found reduced B cell but preserved T‐cell proportion in the lungs of aged mutant mice compared with WT controls (Table 1). The aged mice also displayed reduced B‐cell proportion in the bone marrow and spleen compared with WT aged mice. By contrast, the aged mutant mice showed increased T‐cell proportion in the bone marrow and spleen compared with WT mice. There was also a marked reduction in NK cells in the mutant bone marrow and spleen. These findings suggest that both systemic‐ and organ‐specific changes in adaptive immunity accompany IL10 deficiency. The reduction in B cells in the lung is consistent with known compartmental effects of IL10 (Itoh & Hirohata, 1995; Lee et al., 2002). Others have not shown reduced B cells in the bone marrow of young IL10‐deficient mice potentially implicating an age‐associated phenotype (Kuhn et al., 1993).

**Table 1:** Flow cytometry of lymphocyte subsets

	%B cells	*SEM*	%T cells	*SEM*	%NK cells	*SEM*
Lung						
WT	22.6	2.8	28.5	3.1	3.9	0.6
Mut	8.9*	2.8	28.5	3.2	3	0.6
BM						
WT	15.5	1.6	8.3	0.9	3.6	0.5
Mut	9.8*	3.5	25*	4.9	2.7*	0.5
Spleen						
WT	35.8	5	24.2	2.7	23.5	5
Mut	28.9*	6.4	33.9*	6	1.9*	0.5

Note

*N* = 4–5 aged mice per genotype. Methods noted in Table S1.

* *p* < .05 compared with WT.

**Figure 6:**
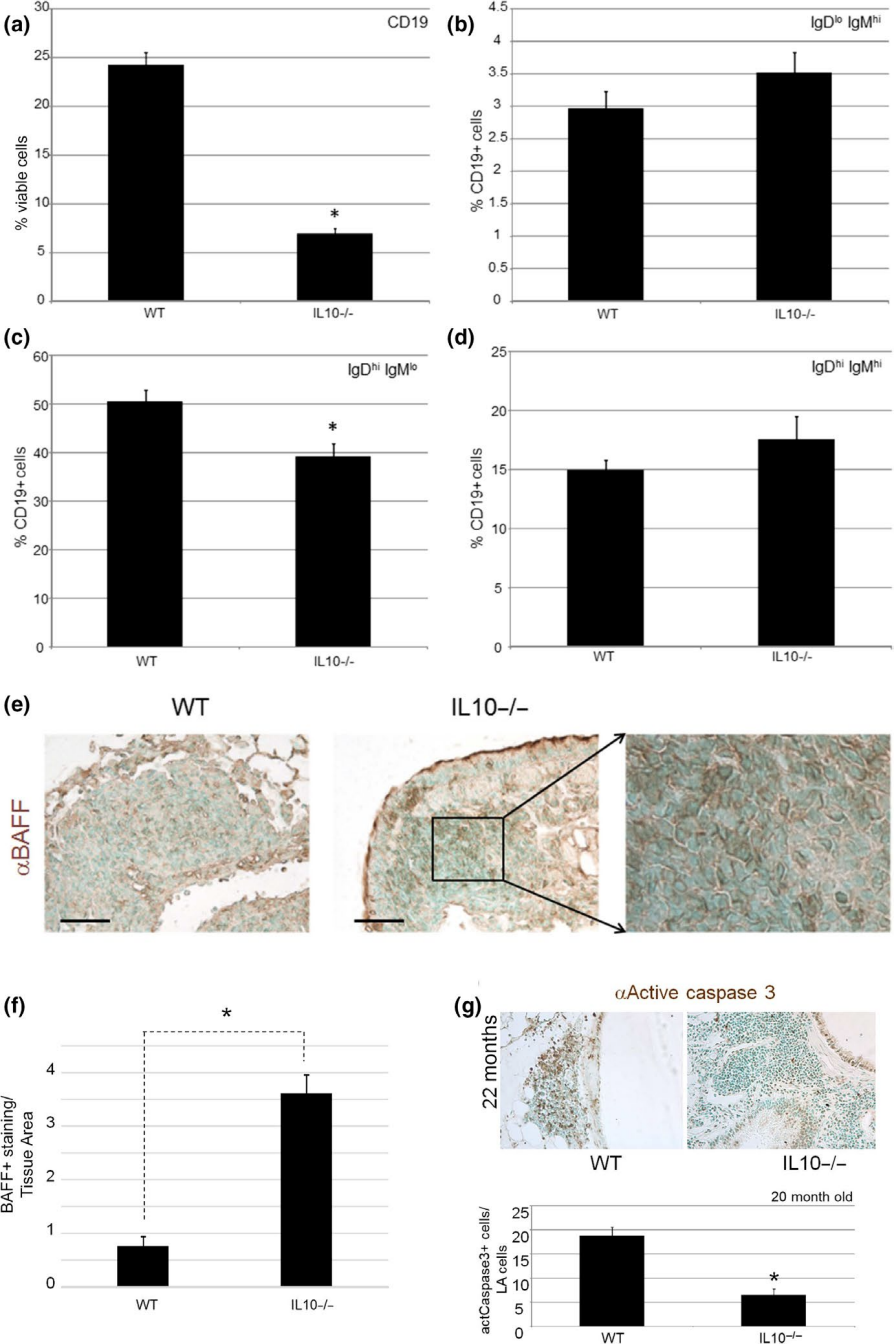
Altered B‐lymphocyte subset abundance and BAFF expression in aged IL10‐deficient lungs. (a) Lymphocyte proportion by flow cytometry in WT and IL10‐deficient lungs. *p* < .001. (b) Single‐positive igDloIgMhi proportion among lymphocytes in WT and IL10‐deficient lungs. *p* = .288. (c) Single‐positive IgDhiIgMlo proportion in WT and IL10‐deficient lungs. *p* = .029. (d) Double‐positive IgDhiIgMhi proportion in WT and IL10‐deficient lungs. *p* = .361. (e) BAFF IHC in LAs of aged WT and IL10‐deficient mice. (f) Quantitative IHC of BAFF expression in LAs of WT and IL10‐deficient lungs. (g) Active caspase 3 IHC in aged WT and IL10‐deficient LAs. (h) Quantitative IHC of active caspase 3 staining in LAs of WT and IL10‐deficient lungs. *N* = 5–8 mice per genotype and age. *p < .03 compared with age‐matched WT mice

The disparate effects of IL10 deficiency on B‐cell abundance in the LAs versus. the airspace prompted an examination of the B‐cell composition in the aged mutant and WT lung. Since IL10 can have selective effects on B‐cell maturation and lung LAs were increased in IL10‐deficient mice, we used flow cytometry to examine the B‐cell subsets in tissues of aged WT and IL10‐deficient mice. We found a reduction in total B cells (B220+) and mature (IgD+/IgM‐) FO1 B cells in the lungs of IL10‐deficient lungs accompanied by an insignificant trend toward increased immature B cells (IgD−/IgM+) (Figure 6a–d). Recently, the B‐cell survival factor BAFF has been implicated in COPD‐associated lymphoid aggregates (Polverino et al., 2015; Seys et al., 2015). We examined BAFF expression in the LAs and found a significant but paradoxical increase in expression by quantitative immunohistochemistry (Figure 6e,f). To determine whether there was an overall change in cell survival in the IL10‐deficient lung, we performed active caspase 3 staining (Figure 6g,h). We found a 63% reduction in active caspase 3 staining within the aged mutant LAs compared with those of the WT LAs, consistent with their increased number in the mutant lung.

Collectively, these data show a conflation of both inflammatory airspace enlargement and lymphoid aggregate expansion in the IL10‐deficient lung that recapitulates signature features of the COPD lungs and invokes an instructive role for macrophages in alveolar epithelial protection in the aging lung.

## 3 DISCUSSION

We report that IL10 deficiency accelerates the development of aging‐related emphysema and induces progressive lymphoid aggregate formation in the lung. We demonstrate that the systemic loss of the IL10 enhances MMP12 expression in the lung and reduces the macrophage prosurvival effects on alveolar epithelial cells, both of which contribute to the emphysema phenotype. CM from aged but not young macrophages of IL10^−/−^ mice reduce survival and enhance cell death in alveolar epithelial cells and demonstrate attenuated production of the epithelial growth factors KGF and HGF. Supplementation of mutant CM with KGF and HGF recovers epithelial cell survival supporting a critical role of these growth factors in IL10‐mediated alveolar epithelial survival. We also observe an expansion in lymphoid aggregates in aged IL10‐deficient lungs that is associated with reduced cell apoptosis in these structures. The composition of aging‐associated LAs in the lung is altered in the IL10‐deficient milieu with reduced B‐cell content despite enhanced BAFF expression. These findings invoke a previously unrecognized contribution of the cytokine in both the alveolar epithelial and the lung tertiary lymphoid compartments.

Aging‐associated emphysema is a well‐studied pathologic entity that coopts features of both environmental toxin‐induced disease (e.g., cigarette smoke‐induced emphysema) and aging‐associated loss of tissue homeostasis (Calvi et al., 2011; John‐Schuster et al., 2016). The constellation of enhanced oxidative stress and reduced cell survival punctuates aging in multiple tissue beds. IL10‐deficient mice are a well‐accepted model of frailty that permits the interrogation of organ‐specific consequences of the chronic inflammatory frail state (Kane et al., 2016; Westbrook et al., 2017). We show here that late‐onset alveolar enlargement is a feature of this frailty model potentially reconciling a known systemic phenotype of aging with acquired lung disease. Hence, IL10 is a key mediator of aging lung homeostasis.

IL10‐driven macrophage phenotypes contribute to immunosuppressive signaling in a variety of inflammatory states(Chuang, Hung, Cangelose, & Leonard, 2016). How IL10 interfaces with epithelial tissues like the distal alveolar compartment of the lung to promote repair or protection has been elusive given the marginal expression of IL10Ra in lung epithelial cells. We show for the first time that the loss of IL10 increases expression of MMP12, an elastase known to contribute to clinical emphysema. By contrast, IL10 appeared to induce MMP12 production in a macrophage cell line exposed to supernatants from CS exposed B‐cell line (John‐Schuster et al., 2014). Our use of BMDMs, a genetic approach, and an aging context may account for the disparate findings. Here, we show evidence that IL10 can educate macrophages to produce prosurvival factors that enable epithelial survival and repair. Multiple aspects of this finding are of interest. Do certain subsets of lung macrophages exhibit this capability? Adoptive transfer of M1 macrophages in a liver fibrosis model promoted recruitment of endogenous “restorative” macrophages that produced HGF and attenuated fibrosis (Ma et al., 2017). Are there autocrine responses to HGF in the monocyte–macrophage compartment that contributes to repair? Upregulation of cMET, the HGF receptor, occurs with distinct differentiation programs of monocytes (Beilmann et al., 1997; Chen, DeFrances, & Zarnegar, 1996). Additionally, enhanced HGF‐MET signaling in monocyte–macrophages may contribute to anti‐inflammatory effects, possibly via IL10 production (Coudriet, He, Trucco, Mars, & Piganelli, 2010). Clearly, the ability of innate immune cells to support epithelial survival provides a potential bolster against both acute and chronic airspace injuries.

The development of lymphoid aggregates in the lung seems not to reflect a singular type of immune insult but rather represents a coordinated local loss of modulation of both adaptive and innate immune axes. These structures plausibly reflect some form of local injury and variably participate in protection/repair or harm (Richmond et al., 1993; Tschernig & Pabst, 2000). We show for the first time to our knowledge an association between LA formation and emphysema development in a murine model of immunosenescence absent a triggering injury (infection, cigarette smoke, etc.). Whereas increased B‐cell abundance with macrophage expansion accompanies airspace enlargement in COPD patients and aged mice, the findings here show that a distinct immune profile supports the acquired airspace enlargement in the aged IL10^−/−^ lung (Calvi et al., 2011; Hogg et al., 2004; Polverino et al., 2015).

How does IL10 deficiency prime LA maturation? Although the partitioning of B and T cells in LAs is well‐accepted, the assessment of B‐cell subsets has not been carefully performed. We find a strong subphenotype of altered B‐cell maturation and survival in LAs and parenchyma of IL10^−/−^ mice. IL10 is a strong inducer of B‐cell proliferation and activation; thus, its deficiency could affect the composition of B cells in immune structures (Rousset et al., 1992). Since B cells are a major source of IL10 production in lymphoid organs, both autocrine and paracrine mechanisms are likely operative (Madan et al., 2009). The reduced B‐cell abundance in the IL10^−/−^ lung suggests that IL10 either promotes local B‐cell maturation or mediates mature B‐cell migration into the structures. This finding coupled with the fewer mature B cells in the lungs of aged mutant mice implicates the former mechanism as does the increased expression of BAFF, a known B‐cell maturation factor, in the mutant lung. A recent study showed lymphoid aggregate formation in a cigarette smoke‐induced lung injury model (John‐Schuster et al., 2014). Chronic obstructive lung disease, including emphysema, associates with the formation of tertiary lymphoid structures that resemble LAs in the lung parenchyma (Hogg et al., 2004). Increased BAFF expression in the LAs of COPD lungs was recently described and accompanies COPD progression (Polverino et al., 2015; Seys et al., 2015). Whereas the LAs in COPD lungs show expansion of B cells consistent with the prosurvival activity of BAFF, the LAs of the aged IL10‐deficient lungs do not. This finding suggests that BAFF may harbor activities that support LA formation independent of B‐cell expansion.

Limitations in our study include (a) the lack of clinical reagents in relevant cohorts for validation, (b) the lack of identification of the specific macrophage subtypes that are responsible for MMP12 production or growth factor induction, respectively, and (c) the use of immunohistochemical markers alone to establish the cell composition of lymphoid aggregates. Additionally, we have used only a C57Bl/6 background strain rather than multiple inbred strains or collaborative crossed mice. These issues will be the focus of future studies.

In summary, we report that IL10 deficiency results in age‐progressive emphysema and LA development in the murine lung, potentially representing a model of age‐associated COPD emphysema. The finding here that IL10 has a lung homeostatic function in the aged milieu implies that interventions which enhance IL10 production in the aging lung may have some protective role.

## 4 METHODS

### 4.1 Animals

WT and IL10 C57Bl/6 mice were purchased from Jackson Laboratory (Jackson Laboratory, Bar Harbor, ME; National Institute on Aging, Bethesda, MD). Male IL‐10tm/tm and B6 mice were housed in specific pathogen‐free barrier conditions until the appropriate age was reached and then sacrificed. All protocols were approved by the Johns Hopkins University Institutional Animal Care and Use Committee, and all experiments were performed according to the guidelines of this committee.

### 4.2 Isolation and analysis of cells from lungs, spleen, and bone marrow

After organ harvest, lung single‐cell suspensions were obtained as previously described using a combination of mechanical agitation and enzymatic digestion as described (Calvi et al., 2011). For bone marrow, the femur of one leg was flushed with PBS through a 40‐um nylon mesh. Spleens were homogenized with gentle dissection.

### 4.3 Flow cytometry

Cells were extracted from the lungs, bone marrow, and spleen using conventional protocols as above. Details of flow cytometry are provided in the Supplemental Information and prior publication (Calvi et al., 2011).

### 4.4 Quantitative immunohistochemistry

Tissue sections were deparaffinized, rehydrated in an ethanol series, and subjected to epitope retrieval using standard maneuvers (Calvi et al., 2011). Details of the protocol and reagents used are provided in the Supplemental Information.

### 4.5 Tissue morphometry

For histologic and morphometric analyses, mouse lungs were inflated at 25 cm H20 and fixed with 4% paraformaldehyde (PFA) in low melt agarose using standard protocol (Calvi et al., 2011). Details of protocol are provided in Supplemental Information.

### 4.6 Lymphoid aggregate quantitation

Paraffin‐embedded sections of the left lung were stained with hematoxylin and eosin. Lymphoid aggregates in the lungs were identified as dense accumulations of more than 50 cells, quantified, and normalized per 10× field. Ten to 15 fields of the left lung were assessed per mouse.

### 4.7 Alveolar type II cell isolation

Isolation of mouse alveolar type 2 epithelial cells was performed using previously described protocols with modification(Beck et al., 2009; Corti, Brody, & Harrison, 1996; Mercer et al., 2009). Details of protocol are provided in the Supplemental Information.

### 4.8 Macrophage isolation and polarization

Using aseptic technique, femurs and tibia of male and female C57Bl/6 WT and IL10‐deficient mice aged 10–14 weeks following euthanization were collected and placed in dishes containing sterile PBS. The bone marrow was exposed and flushed with sterile PBS using a 2‐cc syringe attached to a 26‐gauge needle. BM cells were cultured in medium containing 25 ng/ml of M‐CSF (PeproTech, Canada) for 7 days. Adherent cells constituting the macrophage population, referred to as BMDMs, were replated and then subjected to different polarization cocktails for 24 hr. The polarization maneuvers were conducted as follows: Cells were treated for 24 hr with LPS (100 ng/ml) and recombinant IFN‐ (10 ng/ml) to induce classically activated (M1) macrophages and with recombinant IL‐4 (20 ng/ml) and IL‐13 (50 ng/ml) (PeproTech Canada) to induce alternatively programmed (M2) macrophages. Polarization of macrophages toward the M1 and M2 phenotypes was established by assessing iNOS, amphiregulin, and arginase expression in the polarized cells.

### 4.9 Conditioned media studies

CM from freshly differentiated BMDMs were diluted at 1:2 with culture media and applied to isolated AECII cells from young or aged mice. After 24 hr, cells were harvested for injury studies as below.

### 4.10 Real‐time PCR

Total RNA isolated from cells with TRIzol‐based protocol was treated with DNase and reverse‐transcribed as described (Calvi et al., 2011). Real‐time qPCR performed using TaqMan gene expression assay mix (Applied Biosystems) after reverse transcription of RNA with the High‐Capacity cDNA Archive Kit (Applied Biosystems, Foster City, CA). Gene expression was normalized to glyceraldehyde‐3‐phosphate dehydrogenase (GAPDH) using the threshold cycle for amplification as 2^−ΔΔC^_T_), whereas ΔΔC_T_ = ΔC_T,Control_ − ΔC_T,Target_. The expression levels of target genes were determined in triplicate or quadruplicate from the standard curve and normalized to GAPDH mRNA level. Probes were obtained from Applied Biosystems.

### 4.11 ELISA analysis

Conditioned media from BMDMs treated with IL10 100 ng/ml were subjected to KGF (KGF ELISA kit, RayBiotech) and HGF (R&D Quantikine ELISA) cytokine measurements.

### 4.12 Statistics

Data were analyzed using Sigmastat and Sigmaplot software. Five to ten animals were used per experimental condition and per genotype. Results shown are representative of three or more replicates. Data are presented as means ± *SD*. Two‐tailed Student's *t* test was used for comparison between two groups, and *p* < .05 was considered significant. Comparisons among three or more groups were performed with ANOVA followed by Tukey's multiple comparison test, using Sigmaplot software.

## CONFLICT OF INTEREST

The authors declare no conflict of interest.

## AUTHOR CONTRIBUTIONS

AM, DD, MM, and HP conducted most of the experiments. RW and SG performed the tissue harvests. JW provided reagents, supervised studies, and critically reviewed the manuscript. ERN designed and supervised experiments and wrote the manuscript. All authors read and commented on the manuscript.

## Supporting information

Supplementary Material

## Data Availability

The data that support the findings of this study are openly available in Johns Hopkins Data Archive https://doi.org/10.7281/T1/4RZTRN (Malinina et al., 2019).
