# Supplementary material for: IL10 deficiency promotes alveolar enlargement and lymphoid dysmorphogenesis in the aged murine lung
Source: Aging Cell. 2020 Mar 14;19(4):e13130. doi: 10.1111/acel.13130 (PMC7189990; doi:10.1111/acel.13130)
Supplement: Supplementary file 1 — Supplementary Material [file ACEL-19-e13130-s001.pdf]

Supplemental Methods:

Flow Cytometry: For two- to six-color immunofluorescence, single cell suspensions were stained at 4°C using predetermined optimal concentrations of antibodies. Erythrocytes were lysed after staining using FACS Lysing Solution (Becton Dickinson). Dead cells were detected by using LIVE/DEAD Fixable Aqua Dead Cell Stain Kit (Invitrogen Molecular Probes). Isolated cells were stained with anti-B220, anti-CD19, anti-IgM, anti-IgD, anti-CD11b, and anti-CD11c. Phenotypic analysis of cell types by marker profile was performed using antibodies (EBiosciences and BD Biosciences). Anti-IgD-FITC, anti-CD19-PE, anti-B220-PerCP, anti-IgM-PE-C7, anti-CD11b-APC-Cy7, anti-lineage-V450, anti-Ly6c-FITC, anti-MHCII-PE, anti-CD49b-PerCP-eF710, anti-CD3-PE-Cy7, anti-CD19-APC, anti-F4/80-APC-H7, anti-Lu6g-V450. Cells were processed in an BDArias flow cytometer and analyzed using FloJo software (Tree Star).

Quantitative Immunochemistry: Sections were blocked for non-specific binding with 3% BSA and incubated with the primary antibodies for 1 hour at room temperature. For DAB immunohistochemistry, following incubation with the primary antibody overnight at 4°C, slides were washed with PBST, incubated with an appropriate biotinylated secondary antibody (Jackson ImmunoResearch) and developed by using ABC and DAB detection reagents (Vector Laboratories). Sections were counterstained with either hematoxylin or methyl green per standard protocol. For immunofluorescence, sections were then incubated with secondary antibodies at 1:200 for 30 minutes at room temperature (Molecular Probes). Sections were counterstained with 4’,6’-diamidinio-2-phenylindole (DAPI) and mounted with Vectashield hard set mounting medium (Vector Labs). Antibodies were used at the following concentrations: Active caspase 3 1:100 (ABCAM), F4/80 1:100(ABCAM), MMP12 1:700 (ABCAM),, Nitrotyrosine 1:250 (ABCAM), B 220 1:200 (eBioscience) , CD 3 1:100 (Biocare medical), BAFF 1:500 (ABCAM). Quantitative immunohistochemistry was performed by normalizing staining to either total cell count or tissue area as indicated using NIS software (Nikon Instruments).

Tissue Morphometry: The lungs were equilibrated in cold 4% PFA overnight, sectioned and then embedded in paraffin wax. Sections were cut at 5 µm and either stained with hematoxylin and eosin (H&E) or processed for immunohistochemistry. Morphometric measurements were performed on H&E stained sections taken at intervals throughout both lungs. Slides were coded, captured by an observer, and masked for identity for the groups. Ten to fifteen images per slide were acquired at 20x magnification and transferred to a computer screen. Mean chord lengths and mean linear intercepts were assessed by automated morphometry with a macro operation performed by NIS Imaging Software (Nikon Instruments).

IL10 and growth factor treatment: BMDMs and alveolar macrophages were treated with IL10 100ng/ml (BioLegend) for 2, 6, 12 and 24h. Freshly isolated AECII cells were treated with CM plus KGF (R&D) at 20 ng/ml or HGF (R&D) at 100ng/ml.

Cell Injury Studies: Cell survival was assessed by Alamar Blue (ThermoFisherScientific). Cell apoptosis was assessed by active caspase 3 activity (CellEvent, Invitrogen). Oxidative stress was assessed using

DCFDA cellular ROS detection assay kit (ABCAM). All cell studies employed positive and negative control conditions.

MMP12 Activity Assay: Fresh lung lysates were subjected to analysis per standard Sensolyte 520 MMP protocol (AnaSpec). All studies performed in triplicate.

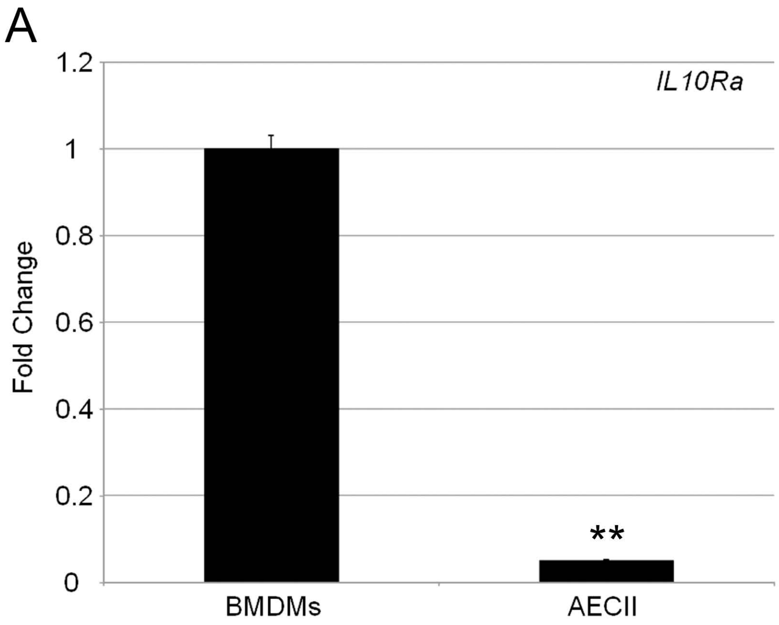

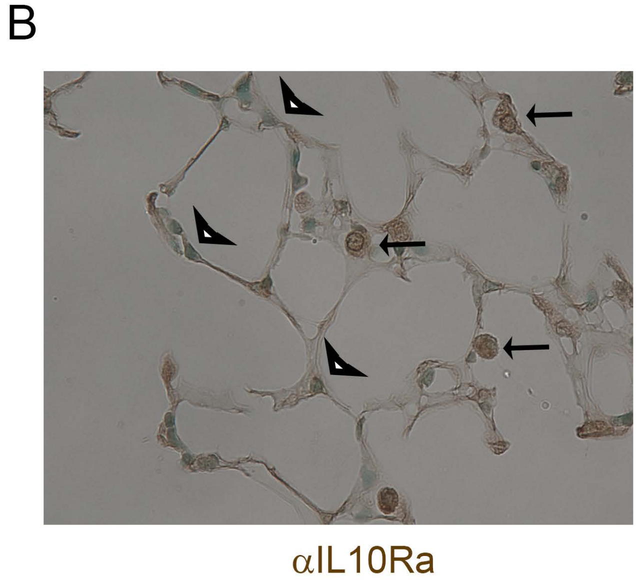

Supplemental Figure 1

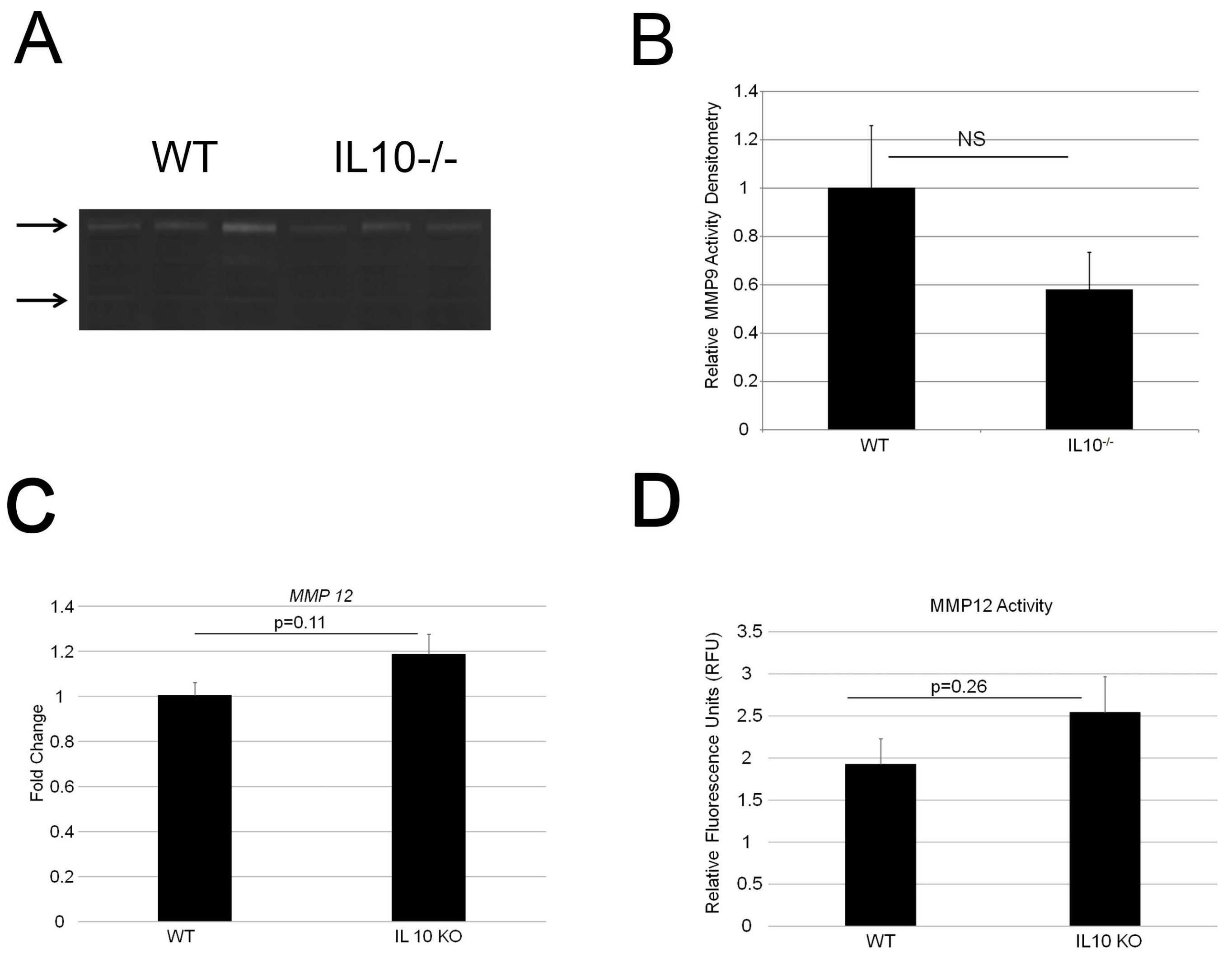

Supplemental Figure 2

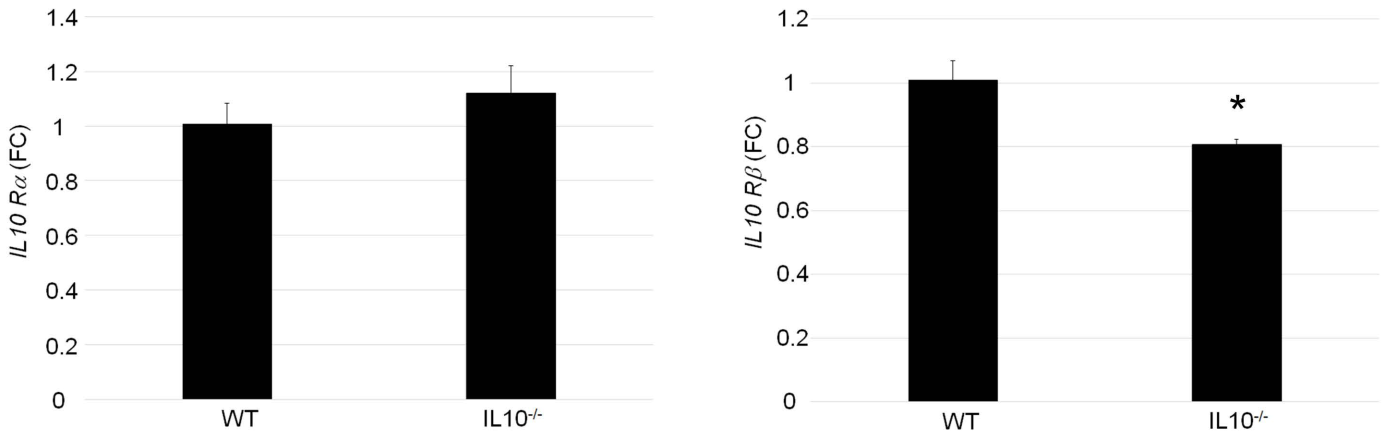

Supplemental Figure 3

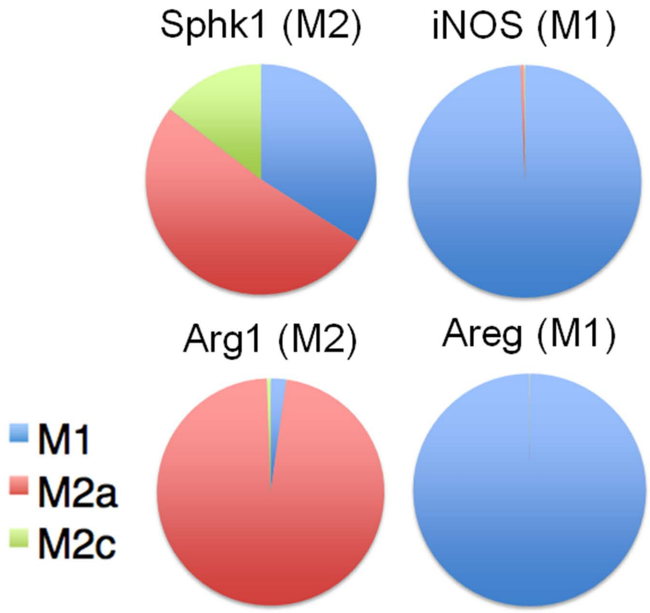

Supplemental Figure 4

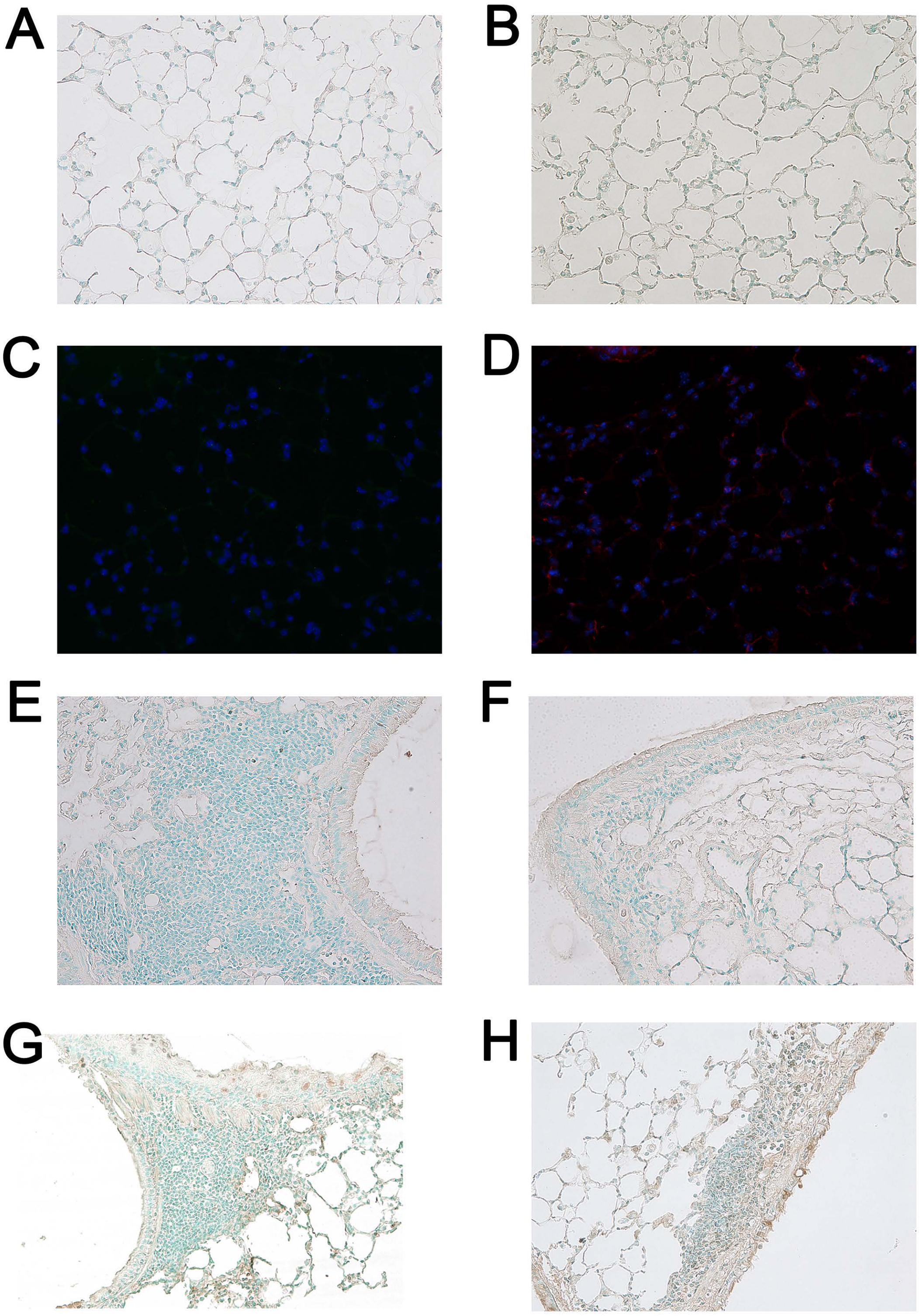

Supplemental Figure 5

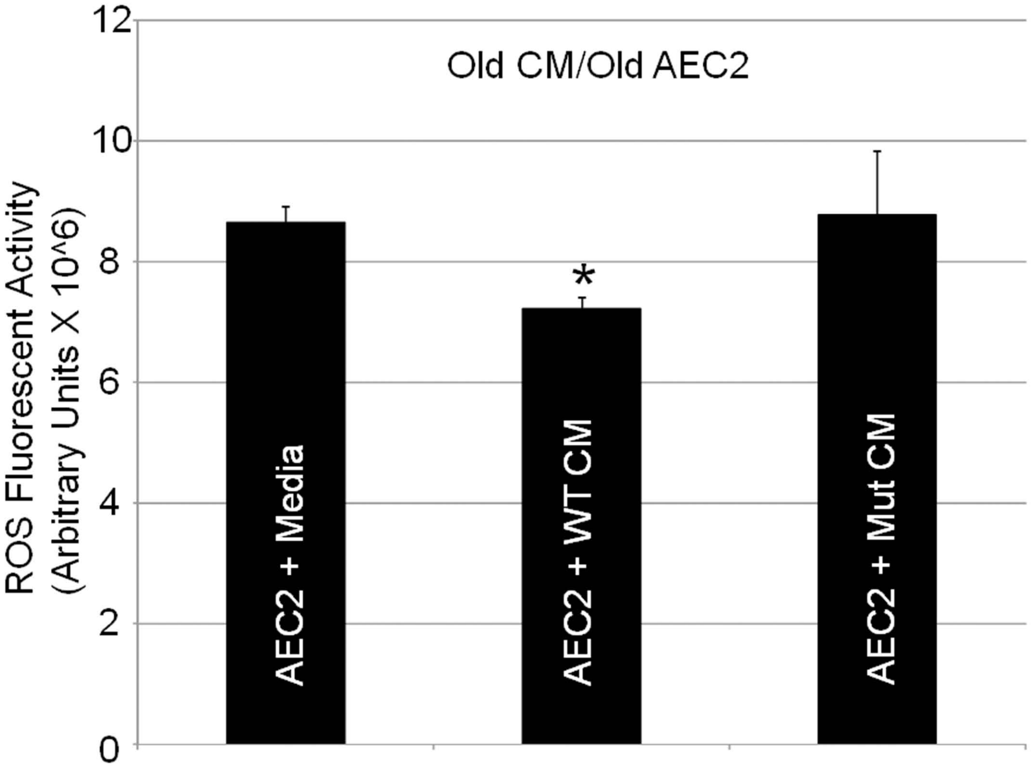

Supplemental Figure 6

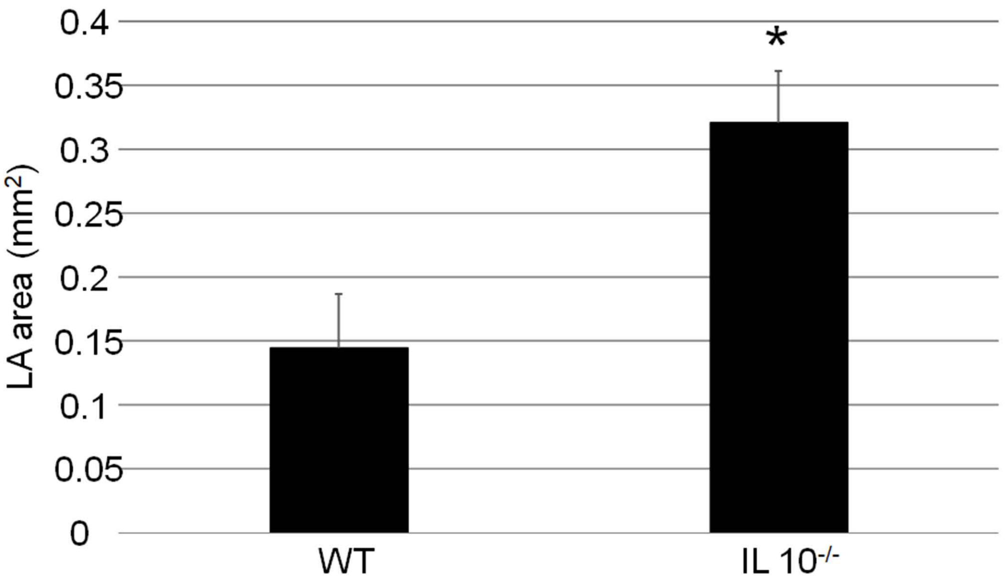

Supplemental Figure 7

WT Lungs

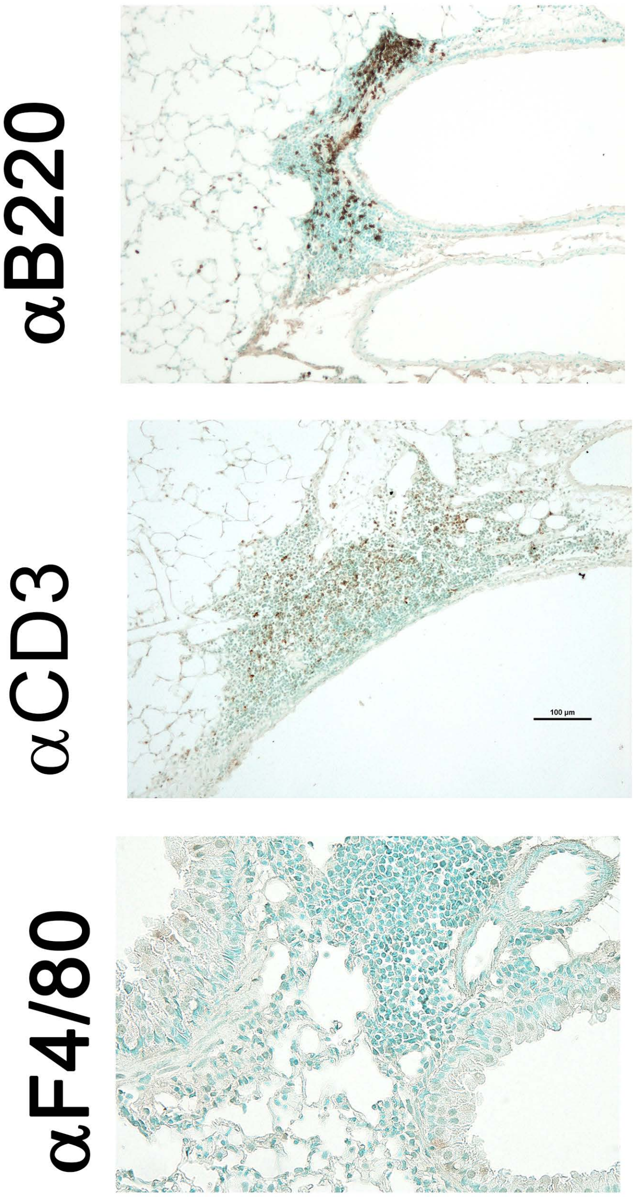

Supplemental Figure 8

Supplemental Data

Supplemental Table 1: Marker Profiles for Flow Cytometry

Supplemental Figure 1: Negligible IL10 receptor expression in WT AECII cells and macrophages. A. Relative expression of IL10Ra by RT-PCR in murine BMDMs and AECII cells. B. Immunohistochemical staining of IL10Ra in WT adult murine lungs. Arrows depict specific expression in alveolar macrophages. Arrowheads show no expression in alveolar epithelial cells.

Supplemental Figure 2: Metalloprotease zymography in WT and IL10-deficient mice. A. Representative zymography of WT and IL10-/- lungs. N=5 mice per age and genotype. B. Densitometric analysis of zymograms of MMP activity in WT and IL10-/- lungs. N=5 mice per age and genotype. C. MMP12 expression in lungs of WT and IL10-/- mice. N=5 mice per age and genotype. D. MMP12 activity in lungs of WT and IL10-/- mice. N=5 mice per age and genotype.

Supplemental Figure 3: IL10 receptor expression in WT and IL10-deficient lungs. Left. IL10Ra expression by qPCR in WT and mutant lungs. Right. IL10Rb expression by qPCR in WT and mutant lungs.

Supplemental Figure 4: Differential expression of macrophage subtype markers in polarized BMDMs (M1, M2a, M2b). Relative proportion of M1 (iNOS and Areg) and M2 markers (Sphk1 and Arg1) expressed by each polarized subtype indicated in diagram. Of note, only M2a cells used in study as M2 cells.

Supplemental Figure 5: Nonimmune control images of immunohistochemical stains. Secondary only staining controls for A. Nitrotyrosine, B. Active caspase 3, C. F4/80, D. MMP12, E. F4/80, F. B220, G. CD3, H. BAFF. All images at 10X magnification.

Supplemental Figure 6: Effect of CM from aged BMDMs on ROS production by aged AEC2 cells. *$p<0.05$ compared with AEC2 cells exposed to media alone. N=5-6 mice per genotype.

Supplemental Figure 7: LA area in aged WT or IL10 deficient mice. N=5-6 mice per genotype. *$p<0.02$ compared with WT mice.

Supplemental Figure 8: Immune cell markers in LAs of WT mice. Representative immunohistochemical staining of LAs in WT aged mice.

Supplemental Table 1: Flow Cytometry Marker Profiles

Cell Population	Marker Profile
**B cells**	CD19+B220+CD3-
ProBcells	CD19+B220-CD3-
MZ	CD19+B220+CD3-$IgM^{hi}IgD^{lo}$
Immature B cell	CD19+B220+CD3-IgM+IgD-
**T cells**	CD3+CD19-B220-
**Macrophages**	F4/80+$Ly6c^{lo}$CD49b+MHCII+
**Monocytes**	Ly6C+MHCII-CD49b-
**Neutrophils**	Ly6g+MHCII-CD11b+CD49b-
**NK cells**	CD11b-/CD19-/CD3-/CD49b+
